# European Society for Organ Transplantation (ESOT)-TLJ 3.0 Consensus on Histopathological Analysis of Pre-Implantation Donor Kidney Biopsy: Redefining the Role in the Process of Graft Assessment

**DOI:** 10.3389/ti.2023.11410

**Published:** 2023-07-04

**Authors:** Gianluigi Zaza, David Cucchiari, Jan Ulrich Becker, Aiko P. J. de Vries, Albino Eccher, Sandrine Florquin, Jesper Kers, Marion Rabant, Michele Rossini, Liset Pengel, Lorna Marson, Lucrezia Furian

**Affiliations:** ^1^ Nephrology, Dialysis and Transplantation Unit, Department of Medical and Surgical Sciences, University/Hospital of Foggia, Foggia, Italy; ^2^ Department of Nephrology and Kidney Transplantation, Hospital Clínic, Barcelona, Spain; ^3^ Institut für Pathologie und Molekularpathologie, University Hospital of Cologne, Cologne, Germany; ^4^ Division of Nephrology, Department of Medicine, Transplant Center, Leiden University Medical Center, Leiden, Netherlands; ^5^ Department of Pathology and Diagnostics, University and Hospital Trust of Verona, Verona, Italy; ^6^ Department of Pathology, Amsterdam UMC, University of Amsterdam, Amsterdam, Netherlands; ^7^ Department of Pathology, Necker-Enfants Malades University Hospital, Paris, France; ^8^ Nephrology, Dialysis and Transplantation Unit, University/Hospital of Bari, Bari, Italy; ^9^ Centre for Evidence in Transplantation, Oxford, United Kindom; ^10^ Department of Surgery, University of Edinburgh, Edinburgh, United Kingdom; ^11^ Kidney and Pancreas Transplantation Unit, University of Padova, Padova, Italy

**Keywords:** kidney transplantation, expanded criteria donors, histopathology, pre-implantation kidney biopsy, consensus paper

## Abstract

The ESOT TLJ 3.0. consensus conference brought together leading experts in transplantation to develop evidence-based guidance on the standardization and clinical utility of pre-implantation kidney biopsy in the assessment of grafts from Expanded Criteria Donors (ECD). Seven themes were selected and underwent in-depth analysis after formulation of PICO (patient/population, intervention, comparison, outcomes) questions. After literature search, the statements for each key question were produced, rated according the GRADE approach [Quality of evidence: High (A), Moderate (B), Low (C); Strength of Recommendation: Strong (1), Weak (2)]. The statements were subsequently presented in-person at the Prague kick-off meeting, discussed and voted. After two rounds of discussion and voting, all 7 statements reached an overall agreement of 100% on the following issues: needle core/wedge/punch technique representatively [B,1], frozen/paraffin embedded section reliability [B,2], experienced/non-experienced on-call renal pathologist reproducibility/accuracy of the histological report [A,1], glomerulosclerosis/other parameters reproducibility [C,2], digital pathology/light microscopy in the measurement of histological variables [A,1], special stainings/Haematoxylin and Eosin alone comparison [A,1], glomerulosclerosis reliability *versus* other histological parameters to predict the graft survival, graft function, primary non-function [B,1]. This methodology has allowed to reach a full consensus among European experts on important technical topics regarding pre-implantation biopsy in the ECD graft assessment.

## Introduction

Kidney transplantation is the first-line treatment for end-stage kidney disease (ESKD), but organ availability does not meet the needs of the large number of potential recipients. For this reason, during the last years, the use of expanded criteria donors (ECD), aged more than 60 years or aged 50–59 years with at least two criteria among hypertension, serum creatinine more than 1.5 mg/dL or death from cerebrovascular accident, has steadily increased [1–3].

Considering the marginal nature of these organs, pre-implantation kidney biopsies have been used to provide a window on the state of the renal graft and it is considered in some settings a valuable decision-making tool as it helps to identify chronic or acute organ damage in order to estimate renal function after transplantation [4–6]. However, in spite of the well-reported clinical utility of this procedure, its use in the daily clinical practice is still debated and poorly standardized.

The role of pre-implantation biopsy in the decision to utilize kidney grafts from ECDs has been somehow controversial: on the one hand, an accurate histological assessment would provide additional information regarding the actual state of a sub-optimal organ, on the other hand the correlation between histological lesions in different compartments (glomerular, tubular, interstitial, vascular) and graft outcome after renal transplantation is not fully understood [5, 7]. Moreover, some histological features may lead transplant centers to discard organs otherwise acceptable based on the Kidney Donor Profile Index (KDPI) or on clinical/functional data [8, 9]. The absence of a clear threshold, as defined by alterations in each compartment of the renal architecture, that accurately predicts an acceptable outcome if the transplant proceeds, makes it challenging to define acceptance criteria based on histological evaluation. In addition, the assessment of pre-implantation kidney biopsies is not standardized in terms of technical procedures and pathologists’ evaluation.

The ultimate goal of the present work is to collect evidence and set up guidelines on the role of pre-implantation biopsy aiming to improve the outcomes and minimize the organ discard: the specific object of this preliminary activity was to reach a consensus about relevant operational procedures as the sampling, processing, staining and reading of the specimens. Currently, no such consensus around pre-implantation biopsy-related technical issues exists, nor does it relate to the impact of histopathological alterations in the different kidney compartments on graft function and survival.

The main reason of this lack of consensus is the difficulty in standardizing the procedure because of different scoring systems, the type of biopsy (wedge vs. needle core), and the differences in reported outcomes. In addition, the pathologists’ expertise has to be taken into account, as it is known to influence the correlation with the outcome [9, 10]. As reported by Azancot et al. [9], donor histology and graft outcome were correlated when the biopsy was evaluated by renal pathologists, but not when they were evaluated by on-call pathologists.

The evaluation of pre-implantation renal biopsies requires specific ultra-specialist training, but in many cases, it is entrusted to an on-call pathologist who often has little knowledge in nephropathology and does not have the opportunity to deal with more expert colleagues [11].

In this context, the possibility of digitizing the slides is essential, allowing for remote evaluation/second opinion [12]. Additionally, the development of digital pathology and modern computerized image analysis tools could also assist the pathologist in slide reading and diagnostic definition [11–13].

All these tools could reduce inter-observer variability, as there is still little agreement among general pathologists, who tend to give higher scores, especially for glomerulosclerosis and arterial thickness, which are the most important parameters for evaluating chronic renal damage [5].

Finally, the employment of pre-implantation kidney biopsy for the evaluation of donor after circulatory death (DCD) is essential [14], but the impact of the specific histological lesions in the Bayesian context of the clinical scenario should be better evaluated.

The deep analysis of the current literature evidence and a peer discussion of all aforementioned issues could help reach a general consensus with a practical clinical impact in kidney transplantation.

For this purpose, in order to develop evidence-based guidance on the standardization and clinical utility of pre-implantation kidney biopsy for the assessment of grafts from ECD, a global panel of four histopathologists, four nephrologists and two transplant surgeons underwent in-depth analysis after the formulation of PICO (patient/population, intervention, comparison, outcomes) questions to develop guidelines on key aspects of the role of pre-implantation histopathology in the process of graft assessment.

After a literature search by the Center of Evidence in Transplantation (CET), the relative statements for each key question were produced, rated according to the quality of evidence using the GRADE approach. The statements were subsequently presented in-person at the kick-off meeting in Prague, discussed and voted [15].

## Methods

The consensus development process was organized by a dedicated Guidelines Taskforce within ESOT and its sections ELITA, EKITA, EPITA, ECTTA, ETHAP, Education Committee, YPT, Transplant International editorial board members and patient representatives. A detailed description of the methodology used was reported previously [15].

Briefly, key issues related to transplantation topic were identified by each working group and specific clinical questions were formulated according to the PICO methodology (PICO, Population, Intervention, Comparator and Outcome) [16]. All PICO questions are listed in Table 1.

**TABLE 1:** List of all PICOs and recommendations.

PICO	Recommendation	Quality of evidence	Strength of recommendation
1. For evaluating chronic lesions in ECD kidneys (P), is the needle core biopsy (I) comparable/inferior/superior to wedge biopsy (C) or punch biopsies in terms of representatively of the entire renal parenchyma (O)?	For the evaluation of chronic lesions in ECD kidneys, needle core and wedge biopsy are both suitable, even though differences may be found in terms of glomerular and vascular assessment. Punch biopsies have potentially similar suitability, although more evidence is required	Moderate (B)	Strong for (1)
2. For the evaluation of chronic lesions in ECD kidneys (P), is the frozen section (I) comparable/inferior/superior to paraffin embedded section (C) in terms of reliability of the reading from pathologists?	For the evaluation of chronic lesions in ECD kidneys the frozen section is inferior to paraffin embedded section in terms of reliability of the reading from pathologists. Frozen sections should not be considered as a first option; however, it could be suitable for use in selected cases such as clinical urgency or other specific contexts	Moderate (B)	Weak against (2)
3. For score assessment of pre-implantation kidney biopsy in the evaluation of ECD (P) is the experienced renal pathologist (I) comparable/inferior/superior to on-call pathologist (C) in terms of reproducibility and accuracy of the histological report (O)?	For score assessment of pre-implantation kidney biopsy in the evaluation of ECD the experienced renal pathologist is superior to non-experienced pathologist in terms of reproducibility and accuracy for the prediction of total parenchyma status	High (A)	Strong for (1)
4. In the quantification of chronic damage in ECD kidneys (P), is glomerulosclerosis (I) more reproducible (O) in comparison with other parameters (interstitial fibrosis, tubular atrophy, wall/lumen ratio, arteriolar hyalinosis) (C)?	In the quantification of the chronic damage in ECD kidneys, glomerulosclerosis is more reproducible in comparison with other parameters (interstitial fibrosis, tubular atrophy, wall/lumen ratio, arteriolar hyalinosis)	Low (C)	Weak for (2)
5. In the quantification of the chronic damage in ECD kidneys (P) is measurement of histological variables with digital pathology (I) comparable/inferior/superior (O) when compared with light microscopy (C)?	In the quantification of the chronic damage in ECD kidneys measurement of histological variables with digital pathology is potentially comparable with light microscopy	High (A)	Strong for (1)
6. In the quantification of the chronic damage in ECD kidneys (P) is measurement of histological variables with the aid of special stainings (Periodic-Acid Schiff, Silver, Picro Sirius Red, Trichrome stainings) (I) comparable/inferior/superior (O) if compared with Haematoxylin and Eosin alone (C)?	In the quantification of chronic damage in ECD kidneys, the use of additional histochemical stainings (including, but not limited to PAS, Silver, Trichrome and/or Picro Sirius Red) is superior to the use of H&E alone in any diagnostic kidney pathology context but can likely not be performed under time constraints in the context of (on-call) organ utilization decision making	Low (C)	Strong for (1) (expert-opinion)
7. In the quantification of the chronic damage in ECD kidneys (P), is glomerulosclerosis percentage (I) more representative than other parameters (interstitial fibrosis, tubular atrophy, arteriolar hyalinosis and cv score) (C) to predict the graft survival, graft function, primary non-function (O)?	Even though no studies are available for head-to-head comparison between GS and the other parameters, the degree of GS in procurement kidney biopsies from ECDs is associated with graft survival	Moderate (B)	Strong for (1)

Following the definition of the PICOs, literature searches were developed by an expert staff from the CET who have expertise in conducting systematic reviews and subsequently integrated, when needed, by the steering committee experts.

The workgroup proposed a recommendation for each key question, based on the quality of evidence rated using the GRADE approach, with high quality rated as A, medium quality as B, and low quality as C; very low quality of evidence was not considered. For evaluating the quality of evidence according to GRADE [15] the following features were considered: study design, the risk of bias, inconsistency, indirectness, imprecision, number of patients, effect, importance and publication bias. The strength of recommendation was rated 1 (strong) or 2 (weak).

Complete information, including the list of consensus conference workgroup domains (and topics noted below), and the process regarding consensus conference participant selection, development and refinement of consensus statements are previously reported beforehand the in-person conference held in Praque, Czech Republic, 13–15 November, 2022 [15].

## Results

After all the methodological steps and two rounds of discussion and voting, 7 statements reached an overall agreement of 100%.

### PICO 1

For evaluating chronic lesions in ECD kidneys (P), is the needle core biopsy (I) comparable/inferior/superior to wedge biopsy (C) or punch biopsies in terms of representatively of the entire renal parenchyma (O)?

#### Analysis of the Evidence for PICO 1

To evaluate chronic lesions in ECD, several techniques are employed, but, to date, no consensus concerning the best procedure for this invasive diagnostic process is available.

A large number of studies, most of them including both ECDs and standard criteria donors, have compared wedge biopsy (WB) *versus* needle core biopsy (NB), demonstrating slight differences. In particular, WB, being more superficial, may provide more glomeruli compared with NB. This may over-estimate the degree of glomerulosclerosis [17–19] and underestimate the extent of the arterial intimal thickening [20]. Different studies also analyzed the correlation between WB or NB and histology of the nephrectomy in the same kidney (Muruve et al., *n* = 9; Mazzuco et al.; *n* = 154) [17, 21] or in the biopsies performed in the early post-transplant period (Bago et al., *n* = 271; Husain et al.; *n* = 392) [19, 22], leading to similar conclusions. Also, two other studies had similar results comparing directly WB *versus* NB in the evaluation of the same organ (Yushkov et al. [23]; Haas et al. [20]). In 226 donors, Yushkov et al. [23] found that optimized needle biopsies were significantly more sensitive in identifying allograft tubulointerstitial scarring as well as intimal fibrous narrowing than WB. However, the technique of NB implied 2 cores of 14-gauge needles. Haas et al. found more severe arteriosclerosis in NB, partly due to the higher number of arcuate arteries in NB compared to WB, but this study was performed in healthy living donors.

Subsequently, Yong et al. [18], demonstrated that WB could be superior to NB in predicting delayed graft function (DGF). However, in this study, the two techniques were not compared in the same patient cohorts and all comorbidities associated with DGF were not considered in the statistical analysis.

Only one study (Bago Horwath et al. [22]), compared punch biopsy (PB) with WB in both pre-implantation and post-transplant biopsies performed for cause within 2 months demonstrated that PB was superior to the other techniques for the diagnosis of Interstitial Fibrosis and Tubular Atrophy (IFTA) and chronic vascular changes.

#### Recommendation 1.1

For the evaluation of chronic lesions in ECD kidneys, needle core and wedge biopsy are both suitable, even though differences may be found in terms of glomerular and vascular assessment. Punch biopsies have potentially similar suitability, although more evidence is required.

Quality of Evidence: Moderate (B).

Strength of Recommendation: Strong for (1).

### PICO 2

For the evaluation of chronic lesions in ECD kidneys (P), is the frozen section (I) comparable/inferior/superior to paraffin embedded section (C) in terms of reliability of the reading from pathologists?

#### Analysis of the Evidence for PICO 2

In a large clinical study, including kidneys in which more than one biopsy was performed [24], authors observed that different procurement biopsies of the same kidney were poorly reproducible (64% of cases, k = 0.14). The correlation between procurement and reperfusion biopsies was also poor, including percentage of glomerulosclerosis, which had 63% agreement (k = 0.15), interstitial fibrosis/tubular atrophy and vascular chronicity, with agreement rates of 82% (k = 0.13) and 80% (k = 0.15), respectively.

A smaller study published by Sagasta et al. [25] found that agreement between observers (on call pathologist *versus* trained pathologist) using the same frozen sections was weaker than the correlation between frozen and paraffin-embedded sections.

Concordance was lower also in the retrospective review of frozen sections (Kendall’s Tau b for Remuzzi score: 0.03), and better in the original report (Kendall’s Tau b for Remuzzi score: 0.67). This comparison revealed that the trained pathologist assigned higher scores when using frozen *versus* paraffin-embedded sections and hypothetically reducing organ acceptance.

Another study [26] showed that frozen and paraffin-embedded sections showed comparable histological changes. Although frozen sections underestimated glomerulosclerosis and arteriolosclerosis and overestimated acute tubular necrosis and interstitial fibrosis those differences were not statistically significant.

Teixera et al. [27] used an aggregate score (MAPI) to assess agreement between frozen sections and paraffin-embedded biopsies, showing improved Kappa coefficient when the total score was used in comparison with the individual parameters. In details, the retrospective review of pathological reports of frozen sections (on-call pathologist) and their corresponding permanent sections (trained pathologist), showed Kappa values ranging from 0.29 to 0.51 for the individual MAPI parameters 0.59 when using the total MAPI score.

#### Recommendation 2.1

For the evaluation of chronic lesions in ECD kidneys the frozen section is inferior to paraffin embedded section in terms of reliability of the reading from pathologists. Frozen sections should not be considered as a first option; however, it could be suitable for use in selected cases such as clinical urgency or other specific contexts.

Quality of Evidence: Moderate (B).

Strength of Recommendation: Weak Against (2).

#### Comment to Recommendation 2.1

In this recommendation, the terms “clinical urgency” was referred to the need to accelerate the transplant procedure due to many factors including very long cold ischemia-time or other logistic necessities.

### PICO 3

For score assessment of pre-implantation kidney biopsy in the evaluation of ECD (P) is the experienced renal pathologist (I) comparable/inferior/superior to on-call pathologist (C) in terms of reproducibility and accuracy of the histological report (O)?

#### Analysis of the Evidence for PICO 3

In a study that included 92 biopsies, 78 kidneys from transplanted and 14 from non-transplanted patients, correlation between the on-call pathologists and the trained pathologist was weak in all the parameters on frozen sections [25]. Trained pathologists assigned higher Remuzzi scores to pre-implantation biopsies from expanded criteria donors than on-call pathologists.

A larger study by Azancot A et al. [9] demonstrated poor to fair agreement for scores generated by on-call and experienced renal pathologists for all histological variables other than glomerulosclerosis, which, conversely, was highly reproducible. In this study, on-call pathologists tended to have higher aggregate scores with a tendency to overcall chronic damage, possibly leading to higher organ discard. It should be highlighted that whilst there was no association between the readings from the on-call pathologist and outcome, evaluation of biopsies by a renal pathologist was significantly and independently associated with estimated 12-month glomerular filtration rate and composite graft outcome.

Subsequently, Girolami et al [5] analyzed the Remuzzi score of 46 discarded kidneys reviewed by three general and two experienced renal pathologists (the original report was blinded) and the intra-class correlation coefficient (ICC) demonstrated that trained pathologists achieved higher values of ICC, reaching excellent or good agreement in most of the parameters, while general pathologists’ values were mainly fair or good.

Notably, the Banff Histopathological Consensus Criteria for Pre-implantation Biopsies endorse a training of general pathologists assigned to donor biopsy evaluation [28].

#### Recommendation 3.1

For score assessment of pre-implantation kidney biopsy in the evaluation of ECD the experienced renal pathologist is superior to non-experienced pathologist in terms of reproducibility and accuracy for the prediction of total parenchyma status.

Quality of Evidence: High (A).

Strength of Recommendation: Strong for (1).

#### Comment to Recommendation 3.1

Based on the literature reports and after our collegial discussion, we recommend, wherever possible, to involve a specialist pathologist for pre-implantation kidney biopsy assessment to minimize the risk of erroneous discard of organs due to the lack of expertise.

### PICO 4

In the quantification of chronic damage in ECD kidneys (P), is glomerulosclerosis (I) more reproducible (O) in comparison with other parameters (interstitial fibrosis, tubular atrophy, wall/lumen ratio, arteriolar hyalinosis) (C)?

#### Analysis of the Evidence for PICO 4

In a study of 44 donor biopsies (50% needle, 50% wedge), glomerulosclerosis (GS), vascular chronicity (cv), tubular atrophy (TA) and interstitial fibrosis (IF) were scored by 3 independent pathologists. The ICCs were 0.87 for GS (the highest), 0.51 for cv, 0.71 for TA and 0.35 for IF. ICC was similar for wedge and needle biopsies [29].

In a more recent study [5], 46 discarded kidneys were identified with their 75 corresponding biopsies (83% wedge and 17% needle). The biopsies were reviewed by three general and two specialist pathologists. Specialist pathologists achieved higher values of ICC with excellent-to-good agreement, while general pathologists’ agreement was fair-to-good. Interestingly, the ICC was highest for GS and was comparable between the general and specialists, whereas ICC for IFTA and vascular changes was poor-to-fair for on-call pathologists and good-to-excellent for experienced renal pathologists. However, the percentage of GS was significantly higher in the biopsies than in discarded organs, demonstrating a “true” sampling error of GS as the majority of biopsies were wedge biopsies.

Using artificial intelligence, a deep neural network segmented normal and sclerotic glomeruli in 98 hematoxylin, eosin and saffron (HES) frozen and 51 formalin-fixed paraffin embedded (FFPE) whole-slide images (WSIs) from 83 donor kidney biopsies, to quantify global glomerulosclerosis. Annotation by three expert pathologists served as the ground truth. A total of 1,544 globally sclerosed and 6,914 non-globally sclerosed individuals were labeled in 149 images. The study demonstrated higher performance of the artificial intelligence model than pathologists. Model accuracy further increased by pooling multiple sections, resulting in a decreased likelihood of erroneous organ discard. However, this study did not compare the reproducibility of GS with other chronic parameters in the biopsy [30].

Two studies from the same center at Columbia University focused on the reproducibility of chronic scores in sequential biopsies from the same donor. Husain et al. [31], included 1,010 cases among which 606 had more than one procurement biopsies. Information about GS, IF, TA, cv was retrieved from the reports. A score from 0 to 3 was assigned for each parameter. Agreement between sequential biopsies reports for kidney that underwent multiple procurement biopsies was evaluated. There was poor overall agreement for the 3 histologic compartments, and agreement was highest for vascular disease and lowest for GS.

More recently, they compared protocol kidney biopsies performed at day 7 and 14 in 69 patients and obtained the reported GS, IFTA, cv and arteriolar hyalinosis scores. Agreement between day 7 and day 14 was best for cv (concordance 78%, k = 0.60). For GS, only a moderate correlation between both time points was found (*r* ^2^ = 0.25) [32].

#### Recommendation 4.1

In the quantification of the chronic damage in ECD kidneys, glomerulosclerosis is more reproducible in comparison with other parameters (interstitial fibrosis, tubular atrophy, wall/lumen ratio, arteriolar hyalinosis).

Quality of Evidence: Low (C).

Strength of Recommendation: Weak for (2).

### PICO 5

In the quantification of the chronic damage in ECD kidneys (P) is measurement of histological variables with digital pathology (I) comparable/inferior/superior (O) when compared with light microscopy (C)?

#### Analysis of the Evidence for PICO 5

The study of Altini et al. [33] detected and classified glomeruli (n: 2,500) in kidney biopsies of 26 subjects using a model based on Convolutional Neural Networks. Global accuracy was higher than 0.98 with precision in classifying healthy and sclerosed glomeruli ranging 0.834–0.935 and 0.806–0.976.

The paper by Bevilacqua et al. [34] tested a Computer-Aided Diagnosis system for segmentation and discrimination of blood vessels *versus* tubules from 10 biopsies in the kidney tissue through the elaboration of histological images: regions of interest identified were in 221:71 vessels and 150 tubules. Results demonstrated that the supervised artificial Neural Network approach was consistent and reveals good performance, after a training phase based on vessels and tubules samples. Accuracy was higher than 0.93, with precision higher than 0.88 in the validation set and higher than 0.91 in the test set.

Luo et al. [35] used donor kidney biopsy WSIs as a source of features in addition to clinical characteristics for graft function prediction, building neural network models to predict stable eGFR and reduced graft function (RGF) in deceased-donor kidney transplant recipients who underwent pre-transplantation biopsy. They tested six prediction models on 219 WSIs. Overall, donor kidney biopsy WSIs were a useful predictor for graft function recovery, showing distinct improvements in the prediction performance of the deep learning algorithm plus the clinical characteristics model. Compared with the clinical data model, the area under the receiver operating characteristic (ROC) curve (AUC) of the clinical data plus the image model for eGFR classification increased from 0.69 to 0.83. Additionally, the predictive performance for RGF increased from 0.66 to 0.80.

In a proof-of-concept study, So et al. [36] reported noteworthy differences in Multiphoton Microscopy derived collagen parameters between donor kidneys with varying KDPI scores. They evaluated the amount (CART) and quality (CRI) of collagen deposition in 20 preimplantation biopsies. Although CART values were identical across all samples, biopsies classified with >85% KDPI demonstrated a significantly higher CART (51.94 vs. 45.61; *p* = .011) than biopsies with 20%–85% KDPI percentages. Conversely, they had lower CRI compared to biopsies with 20%–85% KDPI scores (4.15 vs. 4.53; *p* = .025).

Cascarano et al. [37] collected 26 digital slides taken from the kidneys of 19 donors with Periodic Acid-Schiff staining with the aim to develop a neural network able to detect and classify glomeruli. The workflow allowed the classification of sclerotic and non-sclerotic glomeruli with good performances: 0.99 accuracy, 1.00 precision.

Marsh et al. [38] developed a deep learning model for glomerulosclerosis on a population of mixed wedge and core kidney biopsy cases: 98 frozen and 51 permanent sections. Glomerular counts were compared against annotation ground truth, with accuracy assessed by Pearson correlation coefficient. The model correlated very well with pathologists’ annotations, with a correlation coefficient higher than 0.900.

Salvi et al. [39] developed two models: RENFAST (Rapid EvaluatioN of Fibrosis And vesselS Thickness) for vessels and interstitial fibrosis detection and RENTAG (Robust EvaluatioN of Tubular Atrophy and Glomerulosclerosis) for glomeruli and tubules detection and classification. The RENFAST algorithm is developed and tested on 350 periodic acid–Schiff images for blood vessel segmentation and on 300 Masson’s trichrome stained images for detecting renal fibrosis. In the test set, the algorithm exhibited excellent segmentation performance in both blood vessels (accuracy: 0.8936) and fibrosis (accuracy: 0.9227). The algorithm takes an average computational time 2.91 s against 20 min for pathologist assessment. RENTAG was developed using 61 WSIs for glomerulosclerosis assessment while 22 WSIs were employed for tubular atrophy quantification. The algorithm showed Dice scores of 0.95 and 0.91 for glomeruli and tubules with 100% sensitivity and PPV and little time of computation required.

Eccher et al. [40] evaluated 62 consecutives, previously reported pre-implantation kidney biopsies scanned with the ScanScope Digital Slide Scanner. The slides were assessed for percentage glomerulosclerosis, tubular atrophy, interstitial fibrosis and vascular narrowing using the Remuzzi criteria by two pathologists, one using glass slides and the other using the WSIs viewed on a widescreen computer monitor. After a 2-week washout period, all the slides were re-assessed by the same pathologists using the opposite mode of reporting to that used in the first evaluation. Very high glass-digital intra-observer concordance was achieved for the overall score and for individual grades by both pathologists (κ range, 0.841–0.973).

#### Recommendation 5.1

In the quantification of the chronic damage in ECD kidneys measurement of histological variables with digital pathology is potentially comparable with light microscopy.

Quality of Evidence: High (A).

Strength of Recommendation: Strong for (1).

#### Comment to Recommendation 5.1

Artificial intelligence could potentially help pathologists in their assessment of histological variables in kidney, also reducing interobserver variability. The future potential in terms of 1) infrastructure and organization of care and 2) algorithmic assessment of digital pathology and artificial intelligence needs further evidence.

### PICO 6

In the quantification of the chronic damage in ECD kidneys (P) is measurement of histological variables with the aid of special stainings (Periodic-Acid Schiff, Silver, Picro Sirius Red, Trichrome stainings) (I) comparable/inferior/superior (O) if compared with Haematoxylin and Eosin alone (C)?

#### Analysis of the Evidence for PICO 6

The literature search did not identify articles that fit the search criteria related to the PICO question. Generally, the Scientific Committee strongly beliefs that for any renal pathology setting, only performing an H&E staining is in principle inferior to a dedicated panel of special histochemical staining that also includes Periodic-acid Schiff, Silver, Trichrome and/or Picro Sirius Red stainings.

However, in the setting of (on-call) organ usage decision making specifically, where the optimal decision-making competes with time constraints, processing of special histochemical stains (either performed on frozen sections or fast formalin-fixation protocols) will likely result in an unwanted delay of the organ transplant procedure with a consequent increase of ischemia time for several hours.

#### Recommendation 6.1

In the quantification of chronic damage in ECD kidneys, the use of additional histochemical stainings (including, but not limited to Periodic-Acid Schiff, Silver, Trichrome and/or Picro Sirius Red) is superior to the use of H&E alone in any diagnostic kidney pathology context but can likely not be performed under time constraints in the context of (on-call) organ utilization decision making.

Quality of Evidence: Low (C).

Strength of Recommendation: Strong for (1) (expert-opinion).

#### Comment to Recommendation 6.1

The absence of extensive literature on this topic may not allow for a high quality of evidence, but after discussion, the panel concluded that the strength of this recommendation (expert opinion) was high.

### PICO 7

In the quantification of the chronic damage in ECD kidneys (P), is glomerulosclerosis percentage (I) more representative than other parameters (interstitial fibrosis, tubular atrophy, arteriolar hyalinosis and cv score) (C) to predict the graft survival, graft function, primary non-function (O)?

#### Analysis of the Evidence for PICO 7

In a recent study [41], Stewart et al analyzed a large dataset of 3,851 ECDs recovered in the United States from 2008 to 2012 and reported a significant effect of glomerulosclerosis (GS>10%) on kidney graft survival, even after adjustment for potentially confounding donor and recipient variables. Conversely, the effects of interstitial fibrosis and vascular changes on the outcome were attenuated after adjustment. The BARETO (Biopsy, Anatomy, and Resistance Effects of Transplant Outcomes) study found a clinically and statistically significant effect of GS on 10-year graft survival among ECD kidney transplants. Kidneys having GS>10% were found to have 18% higher risk of graft failure compared with kidneys with GS 0%–5%.

The effect waned beyond 10%, suggesting little or no incremental risk associated with a GS of 20% compared with a GS of 10%. Regarding vascular changes, their data suggest a possible meaningfully large effect of mild-moderate (>25%) or worse vascular changes on long-term graft survival. Interstitial fibrosis seemed to have minimal, if any, prognostic value. These results agreed with those previously published by Anglicheau et al. [42] demonstrating that GS was an independent histological predictor of low eGFR at 1 year and death-censored graft survival. Also, in this case, the cut-off of GS more that 10% was the most significant.

Cheungpasitporn et al. [43] analyzed kidney graft outcomes related to the degree of GS in numerous datasets (>22,000 kidneys) ECDs with a KDPI score >85% from 2005 to 2014. They found that GS >10% is independently related to increased risk of graft loss. Kidneys with >10% GS were associated with 27% higher risk of graft failure compared to kidneys with 0%–10%. Of note, there was no difference in graft survival between 11% and 20% and >20% GS.

These results were in contrast with those previously published by Bodzin et al. [44] using the Organ Procurement and Transplant Network (OPTN) data. Multivariate analysis demonstrated that kidneys from ECDs with 0%–5% GS had no significant differences in graft function compared with those having more than 10% GS.

Additionally, Kayler et al. [45], analyzing a large dataset of kidney transplant recipients (*n*: 597) showed that only the presence of moderate arteriosclerosis and/or moderate arteriolosclerosis (MA), defined as > or = 25% luminal narrowing, was a significant predictor of graft outcome in recipients of ECD kidneys as defined by United Network for Organ Sharing (UNOS) criteria (univariate *p* = 0.02).

Increasing degree of GS in ECD organs was not associated with earlier graft failure in the multivariate analysis (*p* = 0.30).

GS>20% and interstitial fibrosis>25% had a low frequency in the material reviewed, likely reflecting organ use practices and a demonstrable effect on graft outcome could not be demonstrated.

Finally, Sung et al. [46], in another large multivariate analysis performed using the Scientific Registry of Transplant Recipients (SRTR)/Organ Procurement and Transplantation Network (OPTN) data, found that in ECD kidneys, GS was not reliably associated with DGF or graft failure.

#### Recommendation 7.1

Even though no studies are available for head-to-head comparison between GS and the other parameters, the degree of GS in procurement kidney biopsies from ECDs is associated with graft survival.

Quality of Evidence: Moderate (B).

Strength of Recommendation: Strong for (1).

#### Comment to Recommendation 7.1

Based on the aforementioned literature evidence, we cannot draw definitive conclusions regarding the clinical impact of GS in comparison with other chronicity items (IF, TA and cv) to predict graft function and survival. In particular, available studies have only partially considered the quality of the histological interpretation (often performed by non-experienced pathologists), the quality/quantity of the kidney tissue sampling, and the correct data adjustments for demographic and clinical features (e.g., donor/recipient age, recipient’s’ dialysis vintage, HLA matching, comorbidities, immunosuppressive therapy, rate of rejections, infections). No studies have then considered primary non function as the target clinical outcome. Further studies must address these research gaps.

## Summary and Next Steps

This methodology has allowed us to reach a full consensus on important technical topics regarding pre-implantation biopsy in the process of ECD graft assessment and, at the moment, it represents the first attempt in Europe to standardize procedures in this field, including: needle core/wedge/punch technique representatively, frozen/paraffin embedded section reliability, experienced/non-experienced on-call renal pathologist reproducibility and accuracy of the histological report, glomerulosclerosis/other parameters (interstitial fibrosis, tubular atrophy, wall/lumen ratio, arteriolar hyalinosis) reproducibility, digital pathology/light microscopy in the measurement of histological variables, special stainings (Periodic-Acid Schiff, Silver, Picro Sirius Red, Trichrome)/Haematoxylin and Eosin alone comparison in the measurement of histological variables, glomerulosclerosis percentage/interstitial fibrosis, tubular atrophy, arteriolar hyalinosis and intima fibrosis score reliability to predict transplant outcome. Due to the low number of papers published in this field, a main limitation of this consensus is the inclusion of data available from some studies comprising both ECDs and SCDs. However, when possible, we have drawn our conclusions deeply analyzing the specific results referred to ECDs.

We expect that this can have an important clinical impact and represents the basis for the European guideline. In the future, we expect to go into more details on several technical issues and better analyze the relationship of this procedure with the daily clinical practice and hard transplant outcomes, and to review and discuss the role of preimplantation biopsy in ECD kidney acceptance and, ultimately, allocation.

## Workgroup Composition

The ESOT workgroup on Pre-implantation donor kidney biopsy comprised of all main authors, as well as the following panel of independent experts: Klemens Budde, Anthony Dorling, Giuseppe Ietto, Amanda Klein, Georgios Liapis, Maarten Naesens, Mikhail Nozdrin, Stella Stabouli, Stathis Tsiakas, Fabio Vistoli, Angeliki Vittoraki and Oleksii Voroniak.

## Abbreviations

ESKD, end-stage kidney disease; ECD, expanded criteria donors; KDPI, kidney donor profile index; DCD, donor after circulatory death; PICO, patient/population, intervention, comparison, outcomes; CET, center of evidence in transplantation; WB, wedge biopsy; NB, needle core biopsy; DGF, delayed graft function; PB, punch biopsy; IFTA, interstitial fibrosis and tubular atrophy; ICC, intra-class correlation coefficient; GS, glomerulosclerosis; cv, vascular chronicity; TA, tubular atrophy; HES, haematoxylin-eosin saffron; IF, interstitial fibrosis; FFPE, formalin-fixed paraffin embedded; WSIs, whole-slide images; RGF, reduced graft function; ROC, receiver operating characteristic; AUC, area under the ROC curve; RENFAST, Rapid evaluatioN of fibrosis and vesselS thickness; RENTAG, robust evaluatioN of tubular atrophy and glomerulosclerosis; OPTN, organ procurement and transplant network; MA, moderate arteriolosclerosis; UNOS, united network for organ sharing.
